# Data sharing across osteoarthritis research groups and disciplines: Opportunities and challenges

**DOI:** 10.1016/j.ocarto.2022.100236

**Published:** 2022-01-25

**Authors:** Jill Evans, Rebecca I. Hamilton, Paul Biggs, Cathy Holt, Mark T. Elliott

**Affiliations:** a Institute of Digital Healthcare, WMG, University of Warwick, Coventry, CV4 7AL, UK; b Arthritis Research UK Biomechanics and Bioengineering Centre, Cardiff University, Cardiff, CF10 3AT, UK

**Keywords:** Osteoarthritis, Data sharing, Data harmonisation

## Abstract

**Background:**

Osteoarthritis is a heterogeneous condition characterised by a wide variety of factors and represents a worldwide healthcare challenge. There are multiple clinical and research specialisms involved in the diagnosis, prognosis and treatment of osteoarthritis, and there may be opportunities to share or pool data which are currently not being utilised. However, there are challenges to doing so which require carefully structured solutions and partnership working.

**Methods:**

Interviews were conducted with nine experts from various fields within osteoarthritis research. A semi-structured approach was used, and thematic analysis applied to the results.

**Results:**

Generally, osteoarthritis researchers were supportive of data sharing, provided it is done responsibly and without impacting data integrity. Benefits identified included increasing typically low-powered data, the potential for machine learning opportunities, and the potential for improved patient outcomes. However, a number of challenges were identified, relating to: data security, data harmonisation, storage costs, ethical considerations and governance.

**Conclusions:**

There is clear support for increased data sharing and partnership working in osteoarthritis research. Further investigation will be required to navigate the complex issues identified; however, it is clear that collaborative opportunities should be better facilitated and there may be innovative ways to do this. It is also clear that nomenclature within different disciplines could be better streamlined, to improve existing opportunities to harmonise data.

## 1 Introduction

Osteoarthritis (OA) is a heterogeneous condition characterised by a wide variety of clinical factors and is a significant health challenge worldwide.^1^ Subsequently, OA research covers a broad range of disciplines, ranging from cell-based studies through to population level, epidemiological research. This research data is often silo'd and rarely shared outside of individual research groups. This reduces the transparency of the research^1^ and also limits the opportunity for future research projects outside of a specific institution to utilise these datasets. The recognition of data sharing limitations is increasing along with the prevalence of open data initiatives within research communities^2^, with many funding agencies and journal publishers now promoting or requiring the data to be made available in an accessible way. However, the sharing of health and medical research data is often a complex process.^3^ Research across health disciplines outside of OA research (e.g., genomics^4^, cancer^5^ and spinal cord injury^6^) have identified some of the challenges and barriers to data sharing alongside the opportunities. Privacy, consent and ethical approval have been reported as significant barriers to date.^2^^,^^7^ Privacy is a primary concern from a public/patient perspective, with many people wanting reassurances that their data will remain anonymous.^7^ However, this can create challenges where researchers wish to take advantage of data sharing for combining data from different sources or for longitudinal studies where some level of patient identification is required.^4^^,^^7^

To facilitate the integration of datasets, clear governance and standardised protocols are required. Currently, there has been limited success across any health and medical research discipline in achieving this^3^^,^^8^; however, in a clinical sense, a good example of a successfully managed data repository is the National Joint Registry in the UK. This is a database that is used to record a standardised set of variables for every joint replacement surgery in the UK.^9^ As such it has become a valuable resource for clinical research into arthroplasty and other musculoskeletal health conditions. Another success on a much larger scale is the UK Biobank, which is tracking the health of over 500,000 participants, through the collection of imaging, blood, activity and other data.^10^ Importantly, the database can be accessed by any researcher through a relatively simple, but robust, application process.

Despite the challenges, data sharing is recognised to provide new opportunities for large integrated databases that will facilitate the use of advanced machine learning (ML) and statistical methods for identifying new patterns in the data. This could be important for OA research, in determining new sub-types of OA, for example.^11^ An early example of this approach was the Osteoarthritis Initiative project (OAI) which was a large multicenter, long-term study that produced a database of OA data relating to imaging, biospecimens and clinical measures.^12^ Recent advances in imaging techniques, biomechanical analysis, wearable technology and ML, have further broadened the variety of OA datasets^11^, and we envisage that future database platforms should be able to collate data across disciplines and research groups.

In this study, our aim was to investigate the opportunities and barriers of:

i). Sharing research data across the OA research community
ii). Implementing an OA research data repository.

We utilised the expertise within the OATech Network+^13^ to explore the current thinking on this topic. A qualitative thematic analysis approach was used to determine the key themes that emerged from the discussions. There was clear consensus on the opportunities around the use of ML, and that data sharing could be made easier through simple changes to the wording of consent forms and ethical applications. There was less agreement on the idea of a core set of variables every study should collect. The findings from this study will help the OA research community begin to establish a robust framework for data sharing and subsequently developing large scale data repositories for the application of ML and statistical analyses.

## 2 Methods

### 2.1 Study design

This qualitative study interviewed OA focussed researchers from various sub-disciplines (see Table 1). The study was granted favourable review by the University of Warwick Biomedical & Scientific Research Ethics Committee (BSREC) and the Health Research Authority prior to any data collection taking place.

**Table 1:** Participants’ identification number (ID), denoted as IPxx (Interview Participant xx) alongside their research area of expertise and key representative quotes).

Participant ID	Specialism(s)/Research area(s)	Quote
IP01	Physical function from clinical perspective	“Because I've got some long-term cohorts, I understand the need for … sort of long-term data management, that was never an agenda when I was doing things ten years ago and I think it's only experienced researchers are probably coming to this now, people are all starting to twig this is an important thing.”
IP02.a	Biomarkers, biomedical engineering, activity monitoring, gait analysis	“I think there's so many inconsistencies, how people capture the data, the capture rates, the type of data and we don't seem to have any standards or guidelines to say this is the bare minimum.”
IP02.b	Biomarkers, biomedical engineering, activity monitoring, gait analysis	“I think specialist knowledge, I think that's the thing, and it's having the data in the right format for them to use. I think the other thing is when we started doing this there weren't many people that knew about it, we had to train the computer scientists to understand where our data came from otherwise, they didn't use it in the right way. So it's about speaking the same languages.”
IP03	Genomics, proteomics	“I think in OA people are pretty collaborative to be honest, because we know how difficult it is to get tissues in the first place.”
IP04	Pathogenesis, biomarkers, cell therapy	“The MRC have set up the Biobank, haven't they, which is a good exemplar of what can be done, […] You could have a common core that different centres could use, that might be a way to improve it.”
IP05.a	Rheumatology, imaging	“MR images, DICOM images are large, stored on people's routine hospital PACS systems, where they don't have to be anonymised, because only relevant clinicians can access them. But, for research purposes, they would have to be anonymised in a very good system before they could be shared. […] And the problem is, if you strip off all the identifiers, it may adversely affect the image analysis that's done later where certain types of image analysis need to know some things about the sequences.”
IP05.b	Rheumatology, imaging	“Apart from GDPR, it's the issue of what did people give consent for? And most people in their studies weren't thinking five years ahead, or 10 years ahead, or pooling their data with other people. […] This to me is main issue number one, it's how do you get the community to include certain phrases, like you should be providing phrases and we'd say, ‘Put these, make sure these are in your ethics’.
IP06	Genomics	“Within the UK Biobank there are measures relating to the musculoskeletal system, so it's possible to identify individuals that do have osteoarthritis and then do a genetic analysis of those patients […]. But once that's done, that just tells you the genetic signal. The next thing is to go in the lab and try and work out what that genetic signal is doing to gene function.”
IP07	Rheumatology, epidemiology, lifestyle interventions	“There's a whole data preparation step that is complicated. […] when we first did it, it took us, like, over a year to go from the receipt of the raw data to the data ready for analysis, and we've written, kind of, programmes and scripts to make that more efficient, and we have shared those on GitHub and Zenodo repositories so that other people can do that more efficiently than we did, to begin with.”
IP08.a	Biomarkers, population studies	“A core data set might look quite different in a clinical trial of knee OA to hand OA to an observational cohort to a cohort that was designed for predictive modelling, so they may have very different things that they would consider absolutely essential. Or a cohort that doesn't have OA yet to a cohort that already has OA. […] if we're going to say, mandate a core set, […] you have to be really clear what settings you are requiring that in and that is appropriate for all the people you are talking to.”
IP08.b	Biomarkers, population studies	“We can't force people into a model of collaboration, but I think we can provide platforms that help make it easier for people if they want to engage. I think I would probably approach it that way.”
IP08.c	Biomarkers, population studies	“A recent barrier we've had, is just around coding of osteoarthritis in the NHS […] A diagnosis of OA, particularly an early diagnosis, is not well coded […] some of it is about the use of the term and when people apply that term, and some of it is just the heterogeneity around the possible codes of things you might call … “Oh, this person has some knee pain,” to, “They have gonarthrosis,” that's knee osteoarthritis, but a term none of us would ever use but is an ICD-10 code, you know. […] so if you are wanting to search for patients who might be eligible for studies, it's a bit of a minefield and not an efficient way.”

One-to-one interviews were conducted due to varying participant locations and availability, allowing participants to speak freely and individually. The interviews were conducted either in person, at the participant's workplace, by telephone, or via video conferencing. Participants were provided with a participant information leaflet prior to taking part and given the opportunity to ask questions. Informed consent was taken by the researcher, on paper for in-person interviews, and electronically for remote interviews. Interviews lasted up to 1 ​hour, with questions from a semi-structured guide, allowing discussion on pre-determined topics with freedom to elaborate.

### 2.2 Recruitment

Interview participants were purposively sampled based on their professional experience and roles. A total of nine participants were interviewed out of 15 invited to take part. The 9th interviewee's (IP09) expertise was in a field not related to OA; they were interviewed regarding their experience in developing a national database for another health condition. Therefore, their responses are not included in this study. The interview participants' field of expertise and respective quotes are detailed in Table 1. Communications were circulated round the OATech Network+ ​asking for experts across different areas of OA research.

### 2.3 Interview structure

We kept the aim of the interviews relatively broad to allow free and open discussion. The main aims of the interviews were defined as:

• Understand data usage across the research themes
• Identify the potential for data sharing in OA research
• Identify the barriers for data sharing in OA research
• Get an indication of what types of data could be integrated and which combinations are likely to give the best outcomes

Questions relating to each of the aims listed above were defined and are listed in the Appendix A - Supplementary data (mmc 1).

### 2.4 Analysis

A thematic analysis was used from audio file transcriptions with major and minor themes and selected verbatim quotes assessed to illustrate the participants’ agreement or disagreement with them. Each participant was assigned a unique speaker code and identifying information was redacted from transcripts prior to analysis to achieve pseudonymisation. The quotes were thematically collated and assessed as a group to determine overall feedback and level of agreement from participants. Themes were still considered of interest when not discussed by all participants due to variation in professional experience. Some discussion points emerged when deviating from the set questions, due to the semi-structured nature of the questions.

## 3 Results

Five common themes were identified during the interview script thematic analysis around data collection and analysis methods, database sizes and data sharing and research collaboration practicalities. An assessment of these is reported with a selection of impactful participant quotes (Table 1) and remaining quotes of interest within Appendix A - Supplementary data (mmc 2).

### 3.1 Potential for use of machine learning in OA research

Interview results revealed a collective basic understanding and positive opinion of ML within OA with agreement of the time saving and analysis advancement benefits of artificial intelligence. Developing ML tools sensitive enough to reliably test hypotheses in small samples was seen as viable and achievable if trained on larger datasets first. Though potentially beneficial for patient outcomes and commercial companies, concerns also arose around the application of pre-trained algorithms on smaller OA datasets. If not applied carefully and cautiously, ML studies could be underpowered and therefore the reliability and validity of the outcomes could be compromised. Participants strongly agreed collaboration with experts was essential due to the specific knowledge required to tackle complex datasets and extract meaningful insights as well as the lack of existing analysts with combined programming and research skills (Table 1, IP02).

### 3.2 Minimum data collection requirements

There was general agreement from all participants that no formal guidelines or frameworks covering minimum data collection requirements currently exist and this was identified as a key factor in data collection inconsistencies. Some common data collection methods were identified, although participants were not aware of any central resource providing information on used methods (Table 1, IP02.a).

Since most OA research is designed on a study-by-study basis with methods determined by research questions, the importance of these differences remain and core data collection requirements were deemed impractical overall (Table 1, IP08.a).

Also noted was the feasibility of standardised core data could be improved by large organisation management such as research councils. Minimum datasets with a view to post-hoc data linkage and the ability to re-use data was seen positively, particularly for resource-intensive studies (Table 1, IP04).

Difficulties in data pooling due to inconsistencies in clinical data were discussed, alongside potential structural improvements. The nomenclature used within OA research (such as International Classification of Diseases-10) was described as poorly defined with too many variations for effective database searching, likely due to the different paths to diagnosis of OA (Table 1, IP08.c). A framework to streamline the codes used and provide guidance for OA clinicians was suggested as a solution.

### 3.3 Barriers to sharing data

Barriers to data sharing approaches identified included the time consuming and resource consuming requirements for data management/storage, governance and ethical considerations. Also discussed was the risk of diluting or invalidating findings, especially when resources such as the OAI^12^ currently exist. Overall, combining datasets was seen as appropriate if there was a compelling argument for adding impact to findings.

The logistics of data storage was agreed as the biggest challenge, mainly establishing a capable data storage solution and funding to do so, with cloud storage reported as a possible solution to explore. Difficulties to address included data security, accommodating dataset size and OA research readiness for data sharing (Table 1, IP01). The responsibility of data preparation and management was also identified as a key concern.

Imaging data was reported to have its own set of challenges due to file size, formatting and anonymisation requirements. This can result in either reducing the data value by removing key information, or missing elements which would make patient identification possible as well as adding complexity of image transfer protocols to NHS or other research systems (Table 1, IP05.a).

The issue of consent for data sharing arose due to many studies spanning over several years, and the introduction of more stringent research guidelines in the General Data Protection Regulation in the UK^14^ (Table 1, IP05.b). Most participants expressed a willingness from OA patients to share their data if they were given a well-established rationale for doing so, with no noticeable changes reported from updating consent to include data sharing.

There was agreement that the original custodian should be responsible for appropriate governance and security of data collected and stored, as well as their recognition where secondary analysis of data is then achieved. A panel approach was suggested to navigate data sharing challenges and manage access against predetermined guidelines for usage. However, acknowledged, was the significant resource required to facilitate this panel approach with considerable administrative responsibility.

### 3.4 Use of data from databanks and databases

Participants reported a large variation of their own dataset sizes, depending on the nature and aims of the study. Time consuming data collection methods resulted in smaller sample sizes as opposed to questionnaires and routine clinical imaging. There was agreement in the opportunity to increase sample size by accessing existing large databases and pooling collected measures.

Examples of data repositories participants were aware of include the OAI^12^, the Clinical Practice Research Datalink^15^, the Imperial College Healthcare Tissue Bank^16^, REDCap^17^, OpenClinica^18^ and the UK Biobank^10^ as well as institution and research centre specific databases. Participants had varied experiences of these but had strong agreement that they provide a foundation for increasing sample size, improving statistical power and reducing replication of previous work, though the ease of access to these differed with reports of steep learning curves and bureaucracy. Also suggested was their use as an alternative to time consuming randomized controlled trials should the relevant data be available. Suggestions were made to use existing datasets to answer specific questions that support a hypothesis with follow up further analysis, and considered to be cost effective (Table 1, IP06). The processing and preparation of raw data to be used collaboratively was considered a sizable, however, worthwhile task that could benefit future research (Table 1, IP07).

### 3.5 OA research collaboration

Positive attitudes were reported towards OA collaborative research due to the difficult nature of obtaining samples and patient data (Table 1, IP03) as well as difficulties reported due to competitive funding and protecting data, though noted as slowly changing. The difficulties of additional effort to prepare data storage and expenses versus the potentially improved research outcomes were discussed with possible solutions. These included improving communication between research groups, avoiding work duplication and creating frameworks and resource introductions to facilitate connections and dissemination (Table 1, IP08.b).

## 4 Discussion

This study found that there was a shared enthusiasm and willingness to share and analyse OA data in large databases from experts in the field that would enhance research outputs alongside reducing the necessary workload. The key findings are summarised in (Table 2.) Large, integrated datasets with data-driven analyses have previously demonstrated significant benefit and led to advanced approaches in precision medicine, targeting interventions for the specific characteristics of a patient's condition.^19^ This is particularly relevant for OA research which covers a broad range of sub-disciplines, but typically consist of datasets which suffer from small sample sizes.

**Table 2:** Key findings and recommendations resulting from the expert interview thematic analysis that will enable better facilitation of data sharing in OA research.

Key Findings	Recommendations
There was consensus from the experts for using a data-driven approach and existing large databases to enhance OA research and reduce future workloads, though it requires specific knowledge.	Create best practice guidelines for ethical approvals and data protection to enable future data sharing, similar to clinical trial registration protocols.
No experts were aware of any current formal guidelines for OA minimum data collection requirements, likely a key factor in data collection inconsistencies.	Investigate storage and management platforms enabling security and control. This could be through national databases or localised (University) storage facilities.
Large scale OA data sharing would benefit from large organisation governance, such as research councils, and improved clinical classification structures.	Facilitate collaborative opportunities between OA and data science researchers, without enforcing a one-size-fits-all approach.
Barriers identified to sharing data include: - Time and resources required – heavy administrative cost. - Risk of diluting or invalidating findings. - Logistics of storing and managing data securely. - Potential ethical issues.	Provide training and guidance on nomenclature within OA, including clinical codes and terminology, enabling researchers to search and use data from a wider range of sources. Encourage streamlining of terminology where possible to harmonise as many datasets as possible.

The insight we have gathered from OA researchers has provided an overview of the current approaches to data sharing, data harmonisation and collaborative working within the field in the UK as well as the common barriers experienced (Table 2, key findings).

Overall, our results suggest that the ability to have access to datasets that facilitate the application of ML methods is likely to transform OA research through the development of new algorithms and pattern identification previously not possible due to time or resource constraints. The application of ML methods is being applied across numerous disciplines^20^ related to OA research. Therefore, pooling of data within and across disciplines is likely to be advantageous for the progress of data-driven research.

It is clear from the discussions, however, that there are numerous challenges to pooling and sharing datasets. This included data storage, whereby the strict governance, ethical and data protection requirements were highlighted. A recent study of digital health data governance in low- and middle-income countries suggested a four-domain framework for helping stakeholders achieve an appropriate level of data protection.^21^ Salient points raised include the avoidance of person-centric gatekeeping – instead using a committee-based approach for access management and long-term storage strategies and the need to implement a well-defined, documented data structure. This corroborates points raised by participants in our interviews, who had a similar viewpoint in the context of OA data sharing. There are also examples of this approach being successful in the UK in different areas, such as The National Joint Registry^9^ and the Cerebral Palsy Integrated Pathway.^22^

Participants in our study also noted that variance in nomenclature and medical coding can make searching and/or sharing existing clinical and research data challenging and may mean that comparable datasets are missed. Similarly, there are multiple clinical IT systems in place in the UK, and that even within these systems, there are inconsistencies in clinical classifications.^23^ OA is a condition with many routes to diagnosis and this can complicate the pattern of clinical coding – this, in turn, can make searching clinical data more difficult than other conditions. It may not be practical to fully standardise the way OA is coded in research and clinical care, but there is a potential opportunity to create and maintain a training or learning structure for researchers. With a system in place, it may be possible to raise awareness among researchers of the various codes and search terms they can use to identify data and/or patients for trials, and potentially to increase datasets.

Even when a dedicated effort is made to harmonise datasets in OA, challenges remain, particularly when attempting to harmonise data in different languages or using different classifications. Post-hoc harmonisation, whilst still the best option in the absence of access to purposively homogenised data, is time-consuming and may still not yield robust results. We observed concerns from the interviewees about standardising data retroactively and how this might impact validity and reliability. Some level of data pooling was seen as possible where appropriate and where measures align, but where significant effort is required to anonymise or homogenise the data, this was not seen as useful. The European Project on Osteoarthritis (EPOSA) experienced such challenges when attempting to combine data from five multinational longitudinal studies.^24^ The EPOSA study found that the lack of agreement on data collection instruments and procedures between OA researchers was a key factor in the heterogeneity of data and concluded that there is an urgent need for such agreement in order to facilitate pooling of cohort datasets. The researchers felt that longitudinal large-scale pooling is possible, but not while such levels of heterogeneity exist.

## 5 Limitations

Our interviewees were all researchers from the United Kingdom, and therefore, we lacked an international perspective on the subjects covered. However, we suggest the results from the study are relevant to all regions with a well-supported, large research community and a robust data management infrastructure.

Due to limitations on time and resources the study was only able to administer one-to-one interviews and would have also benefited from a/a series of structured focus groups to gain more insight on collaborative opinions. Use of online based surveys and questionnaires, interviews of patients on their opinions of data sharing and early career researchers would have enabled a broader perspective on this concept. The study sought to provide the most valuable opinion information with the resources and time available.

## 6 Conclusion and recommendations

The study identifies key points from a thematic analysis of expert interviews for data sharing within OA research. The results revealed clear agreement from the experts on the benefits of data sharing and facilitating larger databases, with concerns about its realistic implementation into OA research. This includes the considerable resources and logistics required as well as the structural needs and partnership of expert knowledge, summarised in Table 2 with recommendations resulting from the study that will aid the advancement of OA research data sharing. Further study development would benefit from investigation of an OA database template with data that is searchable, can be interrogated, and provides a template for further data contributions. There would also be benefit from investigation of other disease-based databases and guidelines provided that would improve the shared use of OA data (Table 2).

## Authors’ contributions

All authors were involved in the design, set up and recruitment stages of the study. JE collected and analysed the data. JE, RH, CH and MTE wrote the manuscript. All authors read, commented on and approved the final manuscript.

## Declarations

Ethics Approval and consent to participate: The study was granted favourable review by the University of Warwick Biomedical & Scientific Research Ethics Committee (BSREC; Ref: REGO-2019-2360) and the Health Research Authority prior to any data collection taking place.

Availability of data and materials: Data sharing is not applicable to this article as no quantitative datasets were generated or analysed during the current study. Transcripts from the interviews are not publicly available due to them containing potentially identifiable information.

Competing Interests: The authors declare that they have no competing interests.

Funding: This study was funded by the 10.13039/501100000266Engineering and Physical Sciences Research Council (EPSRC) via the OATech Network Plus (Grant Ref: EP/N027264/1). The funders played no role in the design, collection, analysis, and interpretation of results or in writing of the manuscript.
