# Supplementary material for: Data sharing across osteoarthritis research groups and disciplines: Opportunities and challenges
Source: Osteoarthr Cartil Open. 2022 Jan 25;4(1):100236. doi: 10.1016/j.ocarto.2022.100236 (PMC9718296; doi:10.1016/j.ocarto.2022.100236)
Supplement: Multimedia component 1 [file mmc1.pdf]

Data sharing across osteoarthritis research groups and disciplines: Opportunities and challenges

Interview subject areas

Aims of the interviews:

- To get a broader picture of data usage across the themes
- Understand the potential for data sharing in OA research
- Understand the barriers for data sharing in OA research
- Get indicators of what types of data could be integrated and which combinations are likely to give the best outcomes
- To gain opinions from both researchers and clinicians

Researcher questions:

AIM: To get a broader picture of data usage across the themes:

- What is the overarching topic of your research in the context of OA (e.g., genetics, biomechanics)?
- What kind of data do you typically capture in your area of research? What measures/variables?
- How is that data typically used to answer a research question (in the context of OA)? E.g. modelling, comparison to control groups, pre vs post etc etc.
- How large are the datasets, typical number of participants etc)?
- Does the data require specialist software to access? If so, what packages, open source or paid license? Can the data be converted to a universal format, e.g. text output?
- Is there anything you feel you could capture but don't?
    - Why is it not currently captured?
    - How might this be achieved?

AIM: Understand the potential for data sharing in OA research

- Does data sharing amongst research groups already occur in your area of research?
    - If yes, how is this achieved, what systems/structures are in place to facilitate?
- Would your research area benefit from access to larger datasets?
    - How/why not?
- Attitudes towards partnership working/data sharing?
- What do you consider to be the most useful data points in OA in terms of stratification and long term monitoring?
- What are the main barriers to stratification and early prediction in OA?

AIM: Understand the barriers for data sharing in OA research

- Barriers to sharing – commercial, patient/participant or project sensitivity, proprietary systems etc, ethical considerations, robustness of data collection processes or materials
- Have they ever attempted to solve these barriers to facilitate sharing/any ideas for doing so?

- What are the data protection requirements eg length of time kept, security, use agreements etc?

AIM: Get indicators of what types of data could be integrated and which combinations are likely to give the best outcomes

- Are you aware of or ever accessed a biobank or similar? Do biobanks exist with types of data your research area collects?
    - Knowledge of, contribution to, attitudes towards biobanks
- Thoughts on machine learning in the field of OA? Aware of machine learning being applied to this area of research?
- Do you see more benefit from combining homogenous data (i.e. creating larger datasets by combining multiple data of the same type) or heterogenous – data integration across disciplines. If hetero – what other areas should be combined?
- What patient reported outcome measures used – are they deemed useful/reliable?
