# Supplementary material for: Data sharing across osteoarthritis research groups and disciplines: Opportunities and challenges
Source: Osteoarthr Cartil Open. 2022 Jan 25;4(1):100236. doi: 10.1016/j.ocarto.2022.100236 (PMC9718296; doi:10.1016/j.ocarto.2022.100236)
Supplement: Multimedia component 2 [file mmc2.pdf]

Data sharing across osteoarthritis research groups and disciplines: Opportunities and challenges

Types of data collected currently

IP002: "So, we've got simple clinical questionnaire data, so your basics on pain and perceived disability and what you can and can't do and your activity levels, alongside full optical motion tracking data doing… of people with EMG and people doing walking, sit to stand, balance tasks, stairs."

IP007: "That always depends on what question you're trying to answer."

IP007: "Within, kind of, bespoke research studies, it's really driven by what the clinical question is, and what the, kind of, vehicle by which you're collecting the data. […] because we're particularly interested in the methodology by which we collect the data as well, we're often trying to, kind of say well we know that there's this opportunity from this particular type of technology, and we know that there's all these sorts of clinical questions, which of those many opportunities and those questions can we pair together to make kind of a useful combination of this particular problem, can be solved with this particular technology opportunity? So the […] smartwatch study, for example, was a pairing together of the opportunity of collecting self-reported data and physical activity data with that problem in OA that we want to measure both pain and activity at the same time."

IP003: "The big data that I collect is from multi-omics projects so they include NMR or nuclear magnetic resonance metabalomics data, mass spectrometry proteomics data, RNA sequencing data and small RNA sequencing data. So they are sort of global ways of looking at different molecules. [...] So there's lots of big datasets and the datasets can come from not just human tissue but also animal tissues and mainly horse."

IP006: "So how it works is that the samples are actually routinely incinerated, so they're surgical waste, so when a patient has a joint replaced the surgeon will remove some of the bone and the cartilage and some of the other surrounding tissues to make space for the prosthesis to go in, and that material is normally then disposed of. What we do is we ask the patient before surgery if they are willing for us to use those specimens for research and they give informed consent."

IP006: "So what we'll do is we'll generate data that's specific to our disease, so we may use cartilage or synovium tissue from OA patients. We may see a gene behaving aberrantly and that correlates with a genetic signal. We'll then ask, 'Does that gene behave aberrantly in other tissues?' And we can then go to databases and have a search of those, and it may be that it's also behaving aberrantly in thyroid tissue or something like that."

IP006: "We have what's known as in silico analysis which is, you know, using databases in essence, just kind of firming up the discoveries that we've made in the lab in the generation of actual new data. […] It's very straightforward and it's designed that way. [...] It doesn't take long to master the databases and get a lot of important information out of them."

IP005: "We would do two types of trials. So, we work in fields of pharmaco epidemiology, clinical studies and interventional studies. So, I guess in the pharmacoepi, it involves using big databases like CPRD. The clinical trials is of two sorts – investigator initiated or company pharma sponsored trials – and we do both symptom management work and the structure, understanding structural imaging biomarkers. And I use the term 'biomarker' generally to mean imaging rather than wet biomarkers which I don't do much with."

IP005: "We have a strong history in MRI and ultrasound here, and we've done OA work in both MR and ultrasound. A lot of what we're doing in the last few years has been working on the osteoarthritis initiative through the available public MRI dataset."

IP001: "Quite often we set questions round about clinical intervention, so we quite like randomised trials where possible. […] We've got a couple of active RCTs, knee replacement for example, whether one implant performed better than a different implant."

Data sharing across osteoarthritis research groups and disciplines: Opportunities and challenges

IP001: The primary thing we would look at is cohort studies. It's much… you need much less of a definitive primary end point for that but we'll obviously have a research question, you can explore a lot of data at the same time. Outcome questionnaires, so again, joint replacement, we have a large data set […], we've been using it for the last ten years, possibly 15 years, where we've been collecting outcome data before and after hip and knee replacement. So, very quickly you end up with 5,000 patients under each procedure and you can look at things like patient satisfaction, you can look at how BMI or comorbidity influences certain scores and look at the methodology in scores and how the constructs are affected by different sort of comorbid parameters."

IP001: It's not so much a longitudinal over time, we do some long term follow up and the idea of it, it was always a quality assurance project and its very initial outset was just to see what patient scores were like prior to and following surgery. [...] This was about collecting patient data on everyone, that was always the key point, to try and understand who did well and who did badly and try and stratify reasons for who did well and who did badly, but then that kind of grew legs and became an ethically approved project that we got funding for

IP008: we would have quite a lot of clinical data, particularly at the baseline point of the study where we're collecting demographic data, but also almost… I would say usually collected, it's not usually collected for the purpose of the study, but the sort of things that we would examine in clinic, you know, is there fluid in the knee, is there crepitus, is there joint line tenderness, those sorts of things. We will also collect imaging information, and often be doing that for the purpose of the study, often x-ray and MRI are the go-tos. And then we will have additional data, and this is where the data sets get a bit bigger, because at each time point we will be sampling often blood, sometimes synovial fluid, sometimes urine, and then generating biomarker information from some of those.

IP008: It really depends on the study, […] and I think sometimes in the sort of classical, the trials you were talking about, often we would be recruiting people who have very bad, say, symptomatic knee OA, so they are coming into the clinic, seeking treatment from a surgeon or from a rheumatologist, or maybe from their GP and we've identified them at that stage, and they're approached, obviously, given information and decide whether they want to take part. So, that would be the classical situation. Often in the at-risk cohorts, we're actually sort of going out and actually finding people and it may be people who don't have osteoarthritis, but they have had a knee injury, or they are a woman who is perimenopausal, or something like that. We're identifying them as an at-risk group and enrolling them.

IP004: It depends what you're trying to do. In some ways you're at the clinical face and trying to assess treatments and behind that you're trying to bring on new treatments and new developments and then behind that again you're trying to understand other ways of monitoring the disease process… understanding about the biology of it and monitoring treatments and prognostic indicators. So the whole spectrum really of health and disease.

IP004: We know what we want and we gather that information.

Standardised/validated measures used

IP002: "Definitely KOOS and […] visual analogue pain scales, and I think it was the Tegner activity scale."

IP002: "We have a slightly different marker set to other people, and I think within our own sets of data we may have slight differences in the marker sets but how you use it… and then it just depends what inverse dynamics model you put it through to get the data out."

IP007: "For population health studies, [...] questionnaires are a bit crude and poor recall, and so on, but with consumer technology, we have the opportunity of objectively measuring physical activity, and so [...] we collect symptoms on a smartwatch watch face multiple times per day, alongside pulling out raw sensor data from the back of the watch."

IP007: "I mean, I see things like visual analogue scales for things like pain, and then there's patient global assessments, I mean we go to OA, things like the KOOS and so on; disability questionnaires like the HAQ. So, yes, I mean, I don't think, so, very much different from what others will have done there."

IP001: "We've done a lot of work around satisfaction and expectation and they're very simple, single question items. So, you can kind of create your own there but we've been using the same one for a long time which gives a bit of credibility to it and they tend to overarch quite well. [...] We've done quite a bit of exploration around about, you know, "Are you happy with your outcome? Did it meet your expectations?" and you know, various facets of satisfaction. We also, we've used the Oxford knee score since the start of that database, a lot of our trials and projects based around the Oxford hip or knee score. Then we're using other scores as well [e.g] the Forgotten Joint Score. [...]Then things like 85D, we've just done a project looking at the new PROMIS score, the PROMIS general 12 item score."

IP001: "We were using… ideally using validated tools and we're either performing further validation in different cohorts and very often a tool has been developed but not really validated in, say, revision arthroplasty for example, and we might look at that and see is it still relevant in this field, or if that was designed in that language is it still relevant in this language? So, we sort of use the strength of data to make sure that the tools we're using are still reliable in their different contexts."

IP001: "There are issues in terms of responsiveness and ceiling effect with the Oxford scores."

IP001: "There are lots of reasons for using different scores, number of questions, applicability, you know, ease of use for patients and researcher but there are certain ones that always come up. So, Americans use the Knee Society Score. They always do, and there are various issues, methodological issues with that because there are patient report and clinician report elements to it, and a summary score which is really methodologically flawed but it's what they use and you know, therefore everyone else should use it too. […] Or you've got the European camps all use the WOMAC or the KOOS, whereas the Brits tend to focus on the Oxford hip and knee score, because that's what we've used for the last 20 years and even if you think there are maybe some issues with it in terms of, say, sensitivity to change, actually you can compare to all of this massive literature, databases out there already and see how you're sitting compared to historical or other parameters. There's a lot of strength in using the same thing repeatedly."

IP001: "But you have to do a lot of detailed analysis with big data sets to understand the difference. So, you're interested in the effects side of these tools, oh, that's quite interesting but that's a statistical methodology and it requires hundreds of patients before and after to make that kind of evaluation. So, anything involving a questionnaire, so a population score, you have to get large numbers, and when I say large I mean modestly large numbers, in the hundreds, to make any sort of meaningful comment on the quality of the score and how it's changing because your sensitivity analysis would be on patients that have certain known factors that influence the score. Back to the Oxford knee score, we know younger patients report slightly better scores. So in a sensitivity analysis, you'd expect to see in your cohort a subtle benefit of being younger in terms of the scores. Females and males report subtly differently, number of comorbidities affect it as well, so when you've got known group comparisons you can check the validity of your response by evaluating how the expected differences are there or not, but again to have some group analysis you've got to have a large enough initial number to make that happen."

How long are data kept?

IP006: “We actually do process our samples relatively quickly and go through them, so they’re not in the freezer for much more than a year or so. But when it comes to things like DNA which you use infrequently, you know, I’ve got samples that are well over a decade old in the freezer.”

Numbers of data held and time/resources to do so – varied across disciplines, for varied reasons

IP002: “We’ve got a database of [...] I think it’s about 200 people, some have imaging as well some don’t. But we’ve got two sets of studies and one the data quality is higher but we don’t have EMG, but we’ve recruited them through MRI so we can access their imaging. We have about 100 controls, a load of patients at different stages and a load of injured, and then we have about 35 where we have recent MRIs linked to their motion analysis.”

IP002: "Actually collecting a database of over 200 people is really quite traumatic so we didn’t worry so much about the follow-ups in many ways. More recently we’ve collected another 35 from this recruiting them from MRI department and that means we can link it, because what we found is we couldn’t prove that our normals didn’t have any signs of OA and we didn’t know exactly how advanced the OA was.”

IP002: “That took about three years, three, four years to collect that. It’s just getting the people in and keeping the lab quality and the time it takes. [...] It’s harder with the older age groups and sometimes with the younger age groups because they have to take time off work to come in, so it’s just… it’s a lengthy process. […] What we find is it takes ages to marker somebody up and get them ready to test, and then the testing doesn’t take that long. […] And of course the labs got to be free and not being used by… there’s this whole load of logistics go into it and there’s always something.”

IP007: “For electronic health records, if it’s primary care data, you can get thousands of participants. […] The CPRD covers a population of about eight million people, and then depending on what population within the general population you’re trying to study will determine how many patients you have. Secondary care data is less widely available for research. We’re accessing our electronic health records from a single local hospital.

- IP003: “So RNA sequencing basically sequences everything in your sample. So you can be trying to map against 20,000 different genes in each of your samples. So they are very large datasets.”

IP003: “The problem with doing omics is it's expensive. So to give you a rough guide, to run ten samples on RNA sequencing, so to look at all the protein coding genes in say ten samples, will cost you about £7,000 depending on where you get it sequenced. So it's expensive. So you normally can't afford to run hundreds of samples.”

IP006: "We get hundreds but we actually do use them all up, so we’re continuously processing these samples and using them in our experiments, so we don’t really have a biobank of tissue specimens, but we do use hundreds of these samples. So we’ll routinely publish studies in which we’ve used 200-300 samples from patients. […] that’s very good when you’re wanting to functionally characterise a gene that you think is implicated in the disease. When you want to identify the DNA polymorphisms themselves that are causing the disease, you have to investigate tens of thousands of samples.”

IP006: “The differences can be relatively subtle, so many two or three percent differences in frequency, and if your sample size is large enough such small differences become highly significant. […] So when you do these genetic screens, which is the beginning of this type of analysis where you’re trying to identify risk polymorphisms, you need tens of thousands of DNA samples. If you’ve found the polymorphism and you think you know which gene it’s targeting, you need several hundred samples from patients to then try and work out what’s going wrong.”

IP008: We're working on some bigger data sets now for the first time, but most of what I've done is in relatively smaller numbers of people, the tens to the hundreds, rather than the thousands.

IP008: I think particularly for the genomics level data, we can't test what we want to test in hundreds of people, we need thousands, and it allows us to search for enough participants that we can actually look at that.

Variance of and approaches towards minimum datasets

IP002: "I think there's so many inconsistencies, how people capture the data, the capture rates, the type of data and we don't seem to have any standards or guidelines to say this is the bare minimum. […] Because if you're going to spend three years collecting data it would be nice to know that it could be used beyond what it was collected for because it's such an expensive thing to have the equipment, to bring the people in, to pay their travel expenses, the researchers' time. It would have been nice that we could have optimised the data set. We had wanted to collect blood and urine for some other studies but that put some people off because they were scared of needles and then the logistics of all of it made it harder, so we ended up not really doing that. So I think it's more just having some core parameters that when you do you record this because that's really important for the analytics people or for linking data sets, I think that's the biggest message we need to get out of some of these things."

IP002: "I suppose what you get a lot with optical tracking is everyone wants their own unique marker set because they all think theirs is better, but it then means that there's lots of data out there that's maybe not quite so easy to cross reference and link together. […] And almost how you then store that data, you know, which bits you want, do you want it post corrections, after… how you store the data because there's so many versions."

IP007: "If you've already got the data collected, if it's not standardised, then your options are either to, kind of, analyse the data from these discrete sources, based on however you've collected them, and perhaps, you know, meta-analyse the results from those studies, if you're trying to answer the second question; or alternatively, mapping them to some sort of common standard, or common data model, that would allow you to then run a unified, kind of, analysis script on various different data sources, but you've mapped them to make them look the same. You've then got the kind of question of, "Well, should we try and standardise what we collect in the first place?" so you then have the opportunity of pooling the data, or alternatively running a single script on the distributed data, but they all happen to be formatted in the same way. I think where you can standardise things, that's useful. It doesn't always make sense to have it all collected in the same way, but where you can, then… and everyone agrees what the standards should be, then that's useful."

IP006: "How it works is that somebody will be tasked with doing a series of experiments, when they're generating the data they'll be interrogated, in a nice way, in meetings. So we'll ask, you know, how robust is the data? What type of analysis have you done, why have you done that? So that tends to be how it works and that's kind of the responsibility of the PI, the principal investigation, because ultimately they're the ones who take responsibility for the published data, so they have to make sure that it's robust and tight and done correctly. And that's just training, it's how we train people as well."

IP008: I think with the natures of our studies, we do have a lot of information, so there will be key things; age, gender, BMI, usually ethnic origin, handedness, footedness, usually, […] certainly in the injury area, most people are using KOOS for knee injury, so I know that's luckily a shared asset across lots of the cohorts.

IP008: as soon as you start bringing in longitudinal factors, people are seeing people at different times and might have done some of these outcomes in different ways.

IP008: a core data set might look quite different in a clinical trial of knee OA to hand OA to an observational cohort to a cohort that was designed for predictive modelling, so they may have very different things that they would consider absolutely essential. Or a cohort that doesn't have OA yet to a cohort that already has OA. […] if we're going to say, mandate a core set, […] you have to be really clear what settings you are requiring that in and that is appropriate for all the people you are talking to, but I wouldn't be against it. […] I think rather than saying it's a mandated, that this is a guideline, that just having considerations about that this is good practice and these are the people who signed up to it, and these are the joint areas and the types of research that this might be relevant to.

IP004: I was thinking that maybe somebody like the MRC could set out key factors, like a core group of properties or information sets that if anybody was collecting samples, if they made sure that they... The MRC have set up the biobank, haven't they, which is a good exemplar of what can be done, so maybe if they set out some key facts and data points that could be collected by each centre collecting samples. You could have a common core that different centres could use, that might be a way to improve it.

IP004: Or just request that if you're collecting samples and patient information, please always collect these things and then you could base your database around that and have other parameters as add ons.

IP004: because you'll want different things for different studies, but if you always had that core group of facts then they could all be on one system regardless of the ancillary datasets if the database was designed correctly

Variance in terminology

IP008: there's different nomenclature that people use, subgroups, phenotypes, subsets, various sort of classifiers from that point of view.

IP008: A recent barrier we've had, it's a perennial thing, actually, is just around coding of osteoarthritis in the NHS. So, if you have a knee replacement, there is a code associated with that, and that's fine. But a diagnosis of OA, particularly an early diagnosis, is not well coded and I think they've multi, that's multifactorial, some of it is about the use of the term and when people apply that term, and some of it is just the heterogeneity around the possible codes of things you might call… "Oh, this person has some knee pain," to, "They have gonarthrosis," that's knee osteoarthritis, but a term none of us would ever use but is an ICD-10 code, you know, to various other sort of things. […] so if you are wanting to search for patients who might be eligible for studies, it's a bit of a minefield and not an efficient way. […] if you're running a study in diabetes or cardiovascular disease, you've got much more efficient ways of searching for people.

IP008: There are about three different primary care systems, and we can't change that, but I think probably having some kind of musculoskeletal framework or osteoarthritis framework that encouraged people to use particular codes, to have some guidance there, use them early and be consistent would be really great.

Data collection in clinical practice

IP007: "For the secondary use of data that's already been collected, we are only able to use whatever's been collected in routine clinical practice. […] we're looking to try and structure the data collection in clinical care so that it's then also more useful for research as well as improving the, kind of, clinical utility of the data that you

collect, but that's quite hard to make those changes within an electronic health record system, particularly for one small specialty within a very large hospital. But we're trying to do that, and getting some progress there.

Use of databanks for research

IP002: "I have shared some totally different type of data for a project and we got there, but the amount of forms we all had to fill in was painful, so I know it's doable. [...] I think people who are wanting to do it have realised the importance but I think that we just have to make sure we set it up with the right governance so that you know people are using… they know how it's been collected and they're using it in an appropriate way."

IP002: "The only thing I have accessed, we have a tissue bank here they set it up for any tissue that's taken clinically so that it can be used for… so there's an overriding ethics that controls it, but they have a process where you apply to them to use the tissue. So you have to use your own ethics but you apply to them to have access to the tissue bank and use some of those tissues, so I think there's people we could learn from in different contexts that have… so we don't reinvent a wheel or find problems that other people have already solved."

IP002: "it has to be there to comply with the Human Tissue Act and all of those things too, so there's all of those things and the ethical [processes] are all set up in such a way that they don't make it too bureaucratic that you have to apply for 15 things before you can take a blood sample and do something with it. So it's been thought out quite well and the governance of it's been thought out quite well."

IP007: "It's a large, complex data set that you have to, kind of, slowly come to understand. […] It is a learning curve with it, like there is with most things and once you understand it, then it becomes that bit more easy to use. I mean it's helpful to have active collaborations with GPs, because sometimes you don't really know… it's not so much about what the codes mean within the system, because you often have, kind of, guidance about what they do mean, but actually how a GP practices. […] If they saw a patient in front of them with this particular problem, how would they go about coding it? Because we're working with the output of what they do in their electronic health records system."

IP007: "There's a whole data preparation step that is complicated. I mean, we've done a lot of medication safety research using CPRD, and when we first did it, it took us, like, over a year to go from the receipt of the raw data to the data ready for analysis, and we've written, kind of, programmes and scripts to make that more efficient, and we have shared those on GitHub and Zenodo repositories so that other people can do that more efficiently than we did, to begin with."

IP003: "If there is data available for... so for my group with ten samples, someone else's group with ten samples, clever people can combine those datasets and then you increase the power of your analysis."

IP003: "I had a student who was a computational biologist who managed to take other datasets online and combine it with my dataset. So my dataset was... it was a small sample size and because of the difficulty in getting different tissues, it was a mixture of sexes. […] So when they combined my dataset with other datasets online they found that the change... age related changes are also dependent on sex. If they hadn't had access to those other papers they couldn't have come to those conclusions."

IP003: "It's normally pretty easy. You do have key words. It might take about half an hour to get your head around it but it's pretty simple to do. […] You just press the buttons and download it."

IP006: So if you're looking for risk factors for osteoarthritis and you're doing a genetic screen you can use subs databases. So the most informative one so far is the UK Biobank.[…] So within the UK Biobank there are measures relating to the musculoskeletal system, so it's possible to identify individuals that do have osteoarthritis and then do a genetic analysis of those patients, and that's already been done and lots of new osteoarthritis risk genes have

been identified through that study. But once that's done, that just tells you the genetic signal. The next thing is to go in the lab and try and work out what that genetic signal is doing to gene function.

IP001: But we will take specific research questions and we'll draw information from the database to answer these questions. […] So I'll conceive a project and I'll go to the data custodian and the data administrator and say, "Right, I want to run this project over the next year or two, I want to collect this data," and we'll get that approved to take part in the database and they'll collect the data for me and then we'll take the data out and we'll run that through analysis."

IP001: "So, it's not a simple thing where anyone can just say, "I want this data and I am going to run these tests." It is still… there is still quite a big process and you have to be working for a public agency or a university as research, you know, to have the infrastructure in the first place to even try and apply for it. So, everything makes a lot of sense in hindsight, it's just like the first time you try and do something there is a lot of new things to learn, but subsequent applications will be a lot more straightforward."

IP008: we have a musculoskeletal tissue bank here and we will tend to use that to acquire tissue samples that would be essentially waste tissue for replacements

IP008: there are maybe two or three different ones, depending on who we're working with and what the nature of the database needed to be, but we've used REDCap quite a bit, I don't know if that helps. OpenClinica as well, so various. But they are essentially bespoke for your study but are sort of open access databases.

IP004: Registries are increasingly important now for studying lots of things. I think they're getting more respect as well. Indeed, they are sometimes suggested as an alternative to clinical trials, because RCTs are incredibly difficult and costly to do.

IP004: we can get access to the full medical notes, if the patients are happy for us to do that. But then we link it to our lab number which is meaningless to anybody else.

Contribution to databases or databanks

IP003: "So when you're publishing big datasets, the sort of datasets that I use, you're publishing a peer review manuscript, it is best practice but depending on the journal not always enforced, to deposit your data on a repository. So for instance if you use sequencing data you could put it on NCBI and you can put a date on that. So if you're worried that people are going to start analysing your data before your paper's out, you can put a date on for a year's time, two years' time to release that data to the public. Now whoever you're depositing your data with will contact you before and say, are you still okay with us releasing your data and if you're not because maybe you've had problems with publishing, you put that back."

IP003: "When you deposit the data there's different columns that you fill out. So like age, sex, species, platform and that sort of thing. So there is a minimum data that you have to put in."

IP003: "it's not very time consuming but for instance it might take you a morning to stick your data on. If you've then… if they're then asking for all these additional things, it might disenfranchise people from putting their data on. But it would be good to have some additional information on the datasets out there, because they aren't there at the moment."

IP003: "The solution is that if you want to publish in a good journal they say you have to publish your data on a repository and they tell you which repository and those repositories produce the highest standards, so would include their end values. You make people know it basically. […] so if you want to put your data in a... your paper in

a good journal then the journal says you have to deposit your data on X, Y or Z repository and those repositories say you have to include this information about your data, that would be the best solution, I think.”

IP003: “I think people need to be aware and I'm not sure how many people are aware. If you're doing these sort of studies all the time then you're obviously aware. The first time I did a paper I didn't realise that you're supposed to put... the day somebody said to me... and the reviewer said, oh you need... you should put your data on a repository. So from then on I did. But until that point... it was my first study, I didn't realise that you were supposed to do that. “

IP003: "Within the OA field it could be MRI datasets, x-ray datasets, all that sort of thing, where there isn't necessarily a repository for you to stick that data. So if that was available and then other people in other fields would be able to collaborate, they'd be able to increase their sample size. […] So if for instance you had the information on the MRI scans of the patients that you'd then taken cartilage from and then done the sequencing, if you could collate all that together, then that would be brilliant.”

IP003: “So if funding bodies said, your data has to be in this repository for everyone to have access to, people would have to do it because it would be part of their funding. […] But in a wider context the Wellcome Trust have got their own online journal, and they stipulate that... and it's free, I think. I've got one or two papers that are on that. But they're completely open. So you have to put all your data, your raw data onto a form that you can just click when you look at the paper, to have access to it. So that is one way that you could do it I guess.”

IP006: “Journals are becoming quite insistent on things like that, and so are the funding agencies. So they will fund a project [...] then they do more or less insist that that data is then made available for others if they want to do secondary analysis. So yes, we do routinely make available our data for third parties. “

IP006: "It’s normally in a format that you’ve created for your own usage anyway, so it’s just a case of uploading it in that format and making it available.”

IP006: “We ourselves are perfectly happy to do it so long as there are means to do it, and that is facilitated principally by the journals.”

IP006: "It’s interesting in a way. The NIH in America, the equivalent of our MRC, has driven a lot of that, so they’ve created a lot of databases. So science is international, so you can often say to a funder or a journal ‘We’ll deposit it on this database.’”

IP005: “We tried early on in the piece to get some sort of national registry of studies for osteoarthritis to understand what people had and it was just on a no funding basis; it was just too hard to develop that dataset. And also, the problem we had when we started to look below the surface of sharing of data and providing combined datasets was ethics.”

IP005: “If we’re going to do something in the UK, it must be different from OAI, it must provide something that differentiates.”

IP005: “You can sign-on and register very quickly, and then if you want lots of images, you have to send them a terabyte disk and they’ll just download all the images onto a terabyte disk and send it back to you. […] Well they used to do that, but it might be you now download it from an FTP site, from a distant site. So, rather than the physical sending, I suspect you download it. And the people I know who work with it are all reasonably happy. It’s like CPRD or any other big database, you have to get used to all the variables, and you know the naming of all the variables, but it’s pretty good. It’s been pretty good to work with.”

IP005: "OAI is free; CPRD has a cost and it takes a lot of expertise to play with it, it’s not something anybody can just take a download. You have to write an application to get in and they look for experience of using stuff. So CPRD covers I think maybe 10% of the UK population currently and has GP life records just about for those.”

IP008: ultimately, I think there's two questions' is well what are you asking, what are you going to find, and how are you going to find it? You know, what's the point? And also, who's going to pay for it? Because that sort of level of databasing and banking in a non-hypothesis driven way is really expensive.

IP008: I can see in some ways the attraction of it and you could put some resource at it, but I don't think anyone, us or anyone else has sort of the current resource to do that unfortunately.

IP008: in theory, it was open to people outside of the university, but I think in practical terms that hasn't really been happening, again, really, just from a resourcing point of view.

IP004: It is happening, it's not the best database and it's always difficult getting everybody's data in the same form to collate something in a common way, but it is there and it is working to some extent. We haven't entered our data into it yet because it would mean a lot of reorganisation of our data ... there's some glitches in their database that would be difficult for us at the moment to comply with. But , we've created our own database

IP004: we would put [our data] onto the ICRS registry already if it was more compatible.

IP004: I think we've got such a good database for what we want from our data that I don't know that we would get anything more at the moment. But like all of these things, they develop with time along and it's a good thing to support. I'd like to support it, but for example, one of the outcome measures that they insist on having, we haven't been using on most of our patients. So you can't go back and take those outcome measures retrospectively.

IP004: If it was more flexible and the outcome measure that we have always used for our patients, our knee patients for example, was a key dataset, then we could put ours on.

Attitudes towards partnership working and collaboration

IP002: "I think the problem is as a group of people interested in arthritis if we all pull together a lot of the time we're saying the same messages but we use a different language or different way, and if we could come forward with a better dialogue that shows that we are all saying the same thing we could be much more effective as a community."

IP002: "I think the problem is when you've done that everyone wants you data and nobody wants to give you credit, because it's a huge amount of work but you don't get that much out of that huge amount of work, if that makes sense. [You don't] necessarily get the credit for how hard it is to clean the data and make sure it's high quality and all that post-processing."

IP007: "Sharing experiences, sharing codes would be useful and we know people don't really do that, and there are reasons why they don't, but also to do with kind of the academic treadmill to an extent, you know, you need to be in competition with others, and so giving away stuff that's taken you a year to do… But then that is in conflict with the transparency that we should have with research, and the, you know, spending the money from research councils and charities efficiently, we should very much should be sharing."

IP003: I think it depends on the person. I review a lot of papers with omics data and people haven't put it in. This is for various levels of journals, you know, from what I would call high impact OA journals, to the less high impact, then people have a... I always make sure that they deposit their data. I basically say I'm not going to accept that

paper for publication unless they do that. But some people are very... they're protective over their data, but I see it as... I'm not funding any of my work personally. […] You could say it's their data. But it's everyone's data and so I don't see any reason why I shouldn't be publishing my data out there unless you've got... you're really protective over it because you think it's got some IP or it's just your personality, you don't want to do that, or you just don't know how to do it."

IP003: “I think in OA people are pretty collaborative to be honest, because we know how difficult it is to get tissues in the first place, to be able to actually do any experiments on. So within the OA field, I found it pretty collaborative.”

IP001: “ I think there are lots of good, especially trial data sets out there, and the thing about the randomised trial, someone has taken it and done an enormous amount of work, it’s taken five, if not ten, years to get the final paper out and there’s a fantastic resource and they’ve written one good paper with it, and that data needs to be packaged and if people could access it, not just for metanalysis purposes but to draw different data and you see that more and more. There are some groups in England are now pooling different data sets to look at certain questions. […] I think the collaboration side of it is still a relatively new thing. So there is obvious resistance to, “Well, I did all of this work and I’m not quite sure how much to package it.””

IP001: I think it’s just very new. You know, usually we go off and we get money and we do a project, [...] and there is an output and that’s great. I might come back and do a secondary analysis of your data at some point and that’s great, and I think the last couple of years, even having to write your data sharing statement for medical journals, you know, it very often says anonymised data may be available from the corresponding author, we have had to think about that internally as well. [...] Money for this trial came from a charity, you know, the data should be available to everyone but what is our logistics for that?” Someone has got to take the mess of a trial data set and package it in a way that can be stored on a university server and then be made available to others when they come with a sensible reason for coming asking for data.” **-**

IP001: “I think it is changing. I do feel that change, there is definitely more willingness to collaborate, definitely. […] I think there’s concerns but they are sensible concerns.”

IP008: my observation there is that there are lots of people doing similar, yet slightly different solutions, some of which are, you know, you can buy, some of which are academically licensed, all of which look a little bit different, maybe have different stage of validity and testing. […] the idea of us all going off and spending our £10,000 developing our app to ask a person X, particularly for patient-reported stuff is challenging.

IP008: I think (a) awareness, so people don’t know what each other are doing. And, (b) the sort of sensible we can do it better, or what we really need to do… it’s not quite right for us, so we’re going to do it again and we’re going to do it better, or it needs to be done in a slightly different way for our study.

IP008: we can’t force people into a model of collaboration, but I think we can provide platforms that help make it easier for people if they want to engage. I think I would probably approach it that way.

Barriers to sharing collected data – appropriate use, governance

IP002: “We need to know that they’re using the data appropriately. I think you don’t necessarily expect to be an author on a paper, but you would expect to see ‘this was…’ the work that collected the data referenced and the acknowledgement that they have shared the data with you, which you don’t always see.”

IP002: “I think there would have to be some sort of panel [...]. So they can apply to a panel that makes sure that you adhere and you can then say ‘yes, you can have this data on these grounds’ and you sign up to it, and there’s all the governance that goes with it. […] Within that you can say, ‘you need to recognise this work and that work with how it was collected’ or whatever. I think there’s a way of doing it administratively that wouldn’t be too burdensome but would allow people to use the data appropriately, and whatever you do with it maybe you have to run it past some committee to make sure you have done that properly.”

IP002: “I think we actually need someone whose responsibility it is to manage it and administer it because I think it’s actually quite hard to do, because everyone comes in and thinks their programme for cleaning the data or processing the data’s better. […] I think at the time we did that study we had a proviso that the data could be shared in future studies as long as it’s anonymised. So it’s making sure we set up future studies in such a way that data can be shared in an anonymised form, or that we can go back to people, follow them up in five or ten years’ time, so that’s one of the things we’re trying to do now. Then we’re looking at the best way to store and back-up data and get our heads round the new Data Protection Act.”

IP007: “we were waiting to publish that before we then shared the data, but we are working out how to share it. We took consent so that we could share it with others, but we haven’t done so yet, but we’re, as I say, working out where it would sit. We want to put it in a… rather than sending out data sets to people, we want to put it within a safe haven and enable access to other people. […] We said that they consented to us sharing it with people where we saw that as being… you know, where that was in our control, rather than taking consent for it to be freely available.”

IP007: "Conceptually, in terms of data sharing, we plan to store the data in a safe haven, or a trustworthy research environment, where we have control over it, there’s an audit trail of who touches the data and what they do to it, there’s a process whereby you can only take things out of the safe haven once it’s been checked by the staff of the safe haven. There would be a data access application process; there would be a review of that application; there’d then be... we have data sharing agreements that people have to sign and sort of terms and conditions of use as well in terms of how the acknowledge the data source, [...] and so on.”

IP005: “Apart from GDPR, it’s the issue of what did people give consent for? And most people in their studies weren’t thinking five years ahead, or 10 years ahead, or pooling their data with other people. […] This to me is main issue number one, it’s how do you get the community to include certain phrases, like you should be providing phrases and we’d say, ‘Put these, make sure these are in your ethics’. Because our problem was, for example, [another University] contacted me recently about a shoulder study we did a decade ago, and we were very happy to share data with them, and they could have had all our patient data. It was an investigator-initiated study and we just went back and were really worried that our ethics would not work and that it was not inclusive enough.”

IP001: “Above all you have to be sure that appropriate data is being safely released, or safely used. I know that in an organisation it is paramount. [...] So, you have got to be very careful, we have got to have processes.”

IP008: we’ve used the software to build a database, basically we have IT people here who help us to do that. We had various solutions over the years, but I’m hoping this is a more sustainable one, because it’s sort of an open access platform that universities can subscribe to. I’m not sure if there’s even an actual cost involved, and for academics you can just use them freely, so it’s quite good. So, it means you can have a secure database for your data that’s hosted in an appropriate place, and it’s compliant with the various things we need to do. So, for example, if we edit things, that that is all tracked and so on.

Barriers to sharing collected data – ethics and forward planning

IP002: “If we could go back to the normative date we’ve collected in the past ten years and follow those people up. It’s getting the permission and the ethics to do that along with those things that with data protection to be allowed to go back and follow people up, even if it’s just an email saying ‘can you tell me if you’ve now got knee problems? […] But having that access to what happens later and to a degree the ability to reassess them would be really valuable, and that’s something we all forget to do or don’t think about. Because you could argue that in ten years’ time I’ll be retired so why would I put that in my ethics? But I think we’ve got to be more about setting the next generation up and about the research that needs to be done rather than a lot of people just think of their own careers.”

IP002: “I think it really varies and I think it’s about getting your PPI involved early on, and most of them if you explain why they get it. […] it’s just having the conversation with people and making sure you express it the right way in your consent things, but so far we haven’t had actually any issue.”

IP006: “We write an ethics that means that we have this specific question in mind but it’s possible that we may have alternative questions in the future and are they agreeable to us using their data and their samples to answer those questions as well. So we write the ethics so that it’s kind of all encompassing.”

IP006: “We’ve noticed that OA patients are delighted that somebody is investigating their disease and wants to know something about it, and when it’s put to them that these samples with basically be burnt or they can go to the lab and people could discover more about your disease but the data that is discovered won’t necessarily come back to you and help you in your treatment, they’re fine, they’re absolutely fine.”

IP005: “I think is an issue within sites and between sites. So, within sites because we often, especially when we set things up 10 years ago, didn’t think we want to come back and dip into things again. We didn’t think about those issues. And sometimes we did, so what are the issues? Ability to re-contact people, it has to be in your consent forms. Ability for the data to be shared with other databases.”

“IP005: “We’ve slowly got better and it’s a bit of our corporate memory about making sure that your in-house patient information sheets are updated and contain some broad statements about further contact, and sharing of data and using broad terms as much as possible. […] But, of course, you have to identify your patients if you’re going to go back, and how did you keep a record of them, why did you keep a record of them when you shouldn’t have after the finish of the study?”

IP001: "GDPR has changed everything. So, what you might have said previously, you know, “We’re going to use your data, anonymised data for research purposes,” you know, tick the box kind of thing, that wouldn’t be enough anymore. So, data that has previously consented under the old framework would be fine, whereas now, the transparency agenda that’s come in really in the last year, the formalisation of that, I think everyone is changing how they tell patients what’s going to happen to their data.”

IP004: It all depends what's in your ethics. We try and go for longstanding ethics; most of ours are for 20 years.

IP004: Because the management is tricky otherwise and you waste valuable samples, which you can do if you haven't got a longstanding ethics, for example if you've only got ethical approval for three years or so for the duration of a particular project. Also, it depends on specific clinical trials; for example if trialing an ATMP, you have to keep the data for a long time, aminimum of 30 years.

Considerations for set-up of shareable data – costs and logistics, storage etc – changes over time and new developments leading to new roles

IP007: “We haven’t yet worked through anything around funding or, kind of, costs for data access, we’re not looking to make a profit, but there may need to be a kind of cost recovery model.”

IP007: " I think that the kind of concept of safe havens is one that’s evolving and I think universities are sort of slowly working out what they’ve got to do and how they support them. Sometimes, I know of instances where there are multiple safe havens in single institutions, so they working out what they do about that as well, is it a physical local storage, or is it stored in the cloud, and again I think this is, kind of, a moving target.”

IP003: “So remember these sets are massive so really you're thinking you're not going to be able to download it onto your computer. You either need to download it into a cloud or into a server at your university. So when the biotechnicians are analysing this data for us, or if we had analysed our data ourselves, you need a big memory in your computer to be able to do that sort of thing, because the datasets are so large.

IP006: “Sometimes there’s collaborations where you share data. Other times what can happen is you may be able to do a piece of analysis that could complement somebody else’s study. So you’ll tend to do it in your own group, you’ll then pass that data onto the other party and they combine that into a larger manuscript, so often it works that way and do that on a regular basis.”

IP006: "When it comes to software issues or anything, that’s all to be ironed out because academics will do that as a matter of course, so they’ll resolve any of those issues, and I think the agencies that support the databases, that’s all fine, I don’t have any particular issues there. I think there may be some issues where if you’ve collected certain biological specimens, for example serum or plasma, then you may use them for a study and then they need to be frozen down and stored, there are sometimes issues over long-term storage, who’s going to pay for that, how retrievable will the samples be and things like that.”

IP006: "say you worked on 200 serum samples from osteoarthritis patients, you would publish the data, make that data available to others but if somebody then wanted to work from those serum samples that’s slightly more arduous because it’s a physical thing. […] And that would be harder to resolve because then you will have issues over ethics and material transfer agreements and all of that kind of stuff, but more difficult. […] I think maybe the UK Biobank is going to override that because they’ve done half a million people, which is a tremendous sum of samples, so that’s enough and we don’t need to worry about these smaller sample collections.”

IP006: “I think the UK Biobank, [...] they come in and hit it really hard with lots of funding, really high sample sizes, organised it extremely well. Sometimes some research projects will be modest in size; they’ll collect specimens, use them for something and then you have a lack of corporate memory. People move on, they forget about them and all this kind of stuff and they’re not particularly retrievable. So it would be good sometimes for smaller samples if there was an entity that ensured that they were brought together and collated and they could be used by others. I imagine there’s a lot of repetition; people will collect the same samples, that in essence somebody’s already done somewhere else.”

IP005: "In terms of can you construct such databases, well the next issue is it’s relatively easy for patient reported outcomes and demographics. Much harder once you get to imaging data and being able to pool and share such data. The big issues are where do you store, DICOM files which are very large. […] And I’m talking that because the majority of the OA research around the country would be using MR, not ultrasound. And MR images, DICOM

images are large, stored on people's routine hospital PACS systems, where they don't have to be anonymised, because only relevant clinicians can access them. But, for research purpose, they would have to be anonymised in a very good system before they could be shared. […] You need special software that strips all identifiers off it. And the problem is, if you strip off all the identifiers, it may adversely affect the image analysis that's done later where certain types of image analysis need to know some things about the sequences. So, it would have to be set up very carefully from the start."

IP005: "You have to have safe site to download things to, and you have to prove that you've got all the data security on your sites before you can get downloads. It's quite laborious and complex. And it takes many months after you take a download before you can clean all the data up and start to do anything with it […] so you need big servers set up to deal with this, and then appropriate software for dealing with big data."

IP001: "Whereas now because I've got some long term cohorts I understand the need for… sort of long term data management, that was never an agenda when I was doing things ten years ago and I think it's only experienced researchers are probably coming to this now, people are all starting to twig this is an important thing."

IP008: I think people would reflect to us that the sort of quality frameworks, these QOFs that the GPs have for other forms of disease, if they had one for osteoarthritis then you'd suddenly get everything lining up, because they would have to code and demonstrate things to get money.

Data harmonisation

IP006: "Well, it's not so much it's homogenising it, it's in science the more evidence you have for something, the more compelling it is. So you tend to combine as much data as you can to show that something genuinely is happening. And the positive side of that is it can get in a more prestigious journal as well. To work in that way you kind of combine data, but you're not homogenising as such, it's providing additional support for a hypothesis."

IP005: "Way back when we started to look at this issue of getting together a UK database for OA studies, it's part, I think, lack of resources, poor ethics and what would we collect? Now there's also never enough studies going on that are collecting things in a systematic way that may make it worthwhile. And are people collecting data better than was collected in the nine-year follow-up of the osteoarthritis initiative, which is freely available now for anybody to use?"

IP005: "My concern would be if we take a whole lot of disparate data bases around the UK, with different non-well controlled imaging acquisition sequences on different magnets, with probably no good quality control, locally about standards of images and pull them together, I think we'd be lucky to come up with a few hundred people. And OAI has got 5,000 people in it and it's all well-controlled."

IP001: "Coming into it there is a lot of new stuff to learn but I think once we get over these sort of teething processes, access to bigger data sets will obviously mean for better studies."

IP008: I sort of sense a bit of maybe naivety is the wrong word, but this idea that, okay, we have all this data and if only we could all put it together, that we would (a) solve OA, and that, (b), that's possible, because I think there is a bit of… There's going be some IP, or at least some ownership around some of this data, when it's clinical data that isn't fully anonymised, it may have not achieved the level of consent for sharing

IP008: you have to have a really good question and have a persuasive reason for people actually putting loads of effort in, because it's quite a pain, the sort of legal side of data sharing. […] You know, is this in the interests of the

research that we set out to do, rather than just, "Oh, let's just chuck this data together and let's hope that something good comes out of it."

IP008: it has to be carefully approached and thought through and there have to be clear analysis plans and data management plans, so you can't do it in a kind of half-baked way. […] you still have to harmonise your data and have the right field names, you can't just do it after, drop everything in, and then sort it out afterwards.

IP008: the coding conversation we had at the start is very pertinent to this, so actually knowing that you have sensitivity and specificity to actually pull out your cases is really critical for this type of project.

Consortia

IP006: "What can happen is sometimes a group of researchers may come together and form a consortium in which they may agree to pool samples, but whether they physically pool the samples or whether they do the analysis on the samples and pool the data, they're kind of slightly different things. […] It would be better perhaps if the samples were pooled centrally in a repository, but sometimes investigators are quite protective of actual physical samples."

IP008: Even that isn't big enough and we're going to have to be working with some larger international consortia, […] there are some really good examples of people working internationally in consortia to answer shared questions.

Machine learning

IP002: "I had a computer analytics person for a couple of years and she did some papers looking at machine code learning and vector analysis. […] So we've used different analytical approaches and tried not to just do the standard just trying to compare time points but use more intuitive machine learning and work with the right computer people, there's not so many of them around."

IP002: "I don't do it, I get someone else in. I think specialist knowledge I think that's the thing, and it's having the data in the right format for them to use. I think the other thing is when we started doing this there weren't many people that knew about it, we had to train the computer scientists to understand where our data came from otherwise they didn't use it in the right way. So it's about speaking the same languages rather than necessarily specialist… I'm sure there is some specialist software but a lot of is their knowledge of what you can do and how you can use that data."

IP007: "It's hard to imagine how you would change, kind of, clinical practice around that, but I think there are emerging opportunities of automated image analysis, so were you to be able to access the raw images and be able to analyse that through what would be a, kind of, a standardised protocol, then you could, kind of, consistently define people as having OA or not. Radiographic OA or not, but that doesn't currently exist, you know, CPRD don't provide you with the raw images, but the… I think the infrastructure around health data research is changing nationally and it may be, in time, that there are image repositories of things that are collected as part of routine care, but that's not close yet."

IP007: "The increasing prevalence of data scientists, which is something that didn't really exist in academic circles certainly ten years ago, that kind of, the recognition of the need for data management, so it's like 90 percent of the

work that we do as researchers, getting the data ready to do the analysis, the analysis part is actually the easy part, but I think as people with those sort of skills are increasingly employed in our sort of health data research departments, then they will bring that knowledge of, and sort of, insight, and the ethos of needing to share these things more openly and widely."

IP003: "It does take a bit of time but it also takes a lot of training to be able to do that. So that's not something I can do but it's something that [a data scientist] as a collaborator was able to do with my dataset, combine it with freely available datasets on the internet."

IP006: "If it does work it could be quite transformative. But it is principally designed to help companies test the efficacy of treatments in a fairly small window of time. Because what tended to happen in osteoarthritis is people progress slowly, companies don't want to do clinical trials of three, four, five years."

IP005: "The numbers in most studies would not be big enough by far. And the problem is, if you look at most x-ray studies of OA, we now know that if you were doing a, even with an enriched cohort, you'd probably need about 600 patients per arm in an x-ray study with a 12-month outcome. And, when you look at most studies, they're 100 patient per arm or 50 patients per arm. […] They're all markedly underpowered. With MR, with cartilage thickness for example, you'd probably need maybe 150 patients, 160 patients in the 12-month period. With bone shape, you might need 100 patients so the more sensitive tools demonstrate structural progressions with smaller numbers.

IP001: "Personalised medicine of course is a fabulously exciting area. You've heard of examples of cancers being effectively cured where medication wasn't working, they've taken immune samples and they have done genetic evaluation of the actual cancer in question and they've pulled out personal factors that will attack that cancer, and they have built them up in culture and then re-inject them back into the patient and that is having a huge effect where that wouldn't on somebody else. You can see how there are potential offshoots to big data approaches, it is just still in its infancy. Where it is all going is of course very interesting"

IP008: that is the risk, that you're just doing multiple testing and then you find things by chance, or if you're coming up with a model to explain your data, it's just horribly overfitted, so i.e. it works perfectly with your little set of data by chance, but it isn't in any way generalisable to anyone else's, and that's the risk. […] Making it bigger is good, but […] sometimes things are different enough, that it doesn't actually help you, that they're better to be dealt with separately. So, one good example, on the injury cohort that we have, which is really interesting because it was recruited in private healthcare, so immediately you just have a slightly different group of people to if you're recruiting in the NHS, they are 70 percent professional sportspeople, so that's really unusual and I appreciate, again, is not the same as if I go down the clinic and sort of recruit 150 people there with knee injury.

IP008: I would think you would probably lose some of the validity of the overall cohort because there would be very big differences with the data sets that you brought in.

IP008: I think talking to statisticians about this is always really insightful because they can often kind of sit back, they might not know the area, but they really get about those sorts of issues that you might not have thought about with your data sets.

IP008: I would probably be in the more sort of conventional statistical camp, and that's because, unless you have absolutely enormous amounts of data, and I mean really big data, and I think hundreds of thousands of cases, perhaps the full biobank level, then I think, again, we probably lack power for some of the things that we think we might be able to do. So, again, you end up with models that aren't really fitted.

IP008: I think there's probably more around diagnosis than there is maybe around prognostic modelling in terms of how solid things look because the system can learn and so on.

IP008: if there was going to be a project here about the very precise language and making sure that's really embedded in what the community understands, so talking… so if you're talking about predictive modelling, making sure that you've got a prognosis researcher to actually look at the language

IP004: It's very useful and it can tell us things that we don't even know about.

IP004: we were training it to use a scoring system which took the radiologist about 35 minutes and of course once you've got the machine learning algorithm sorted, it can be done in a few minutes or seconds.
